# Different Presentation and Outcomes in the Surgical Treatment of Advanced MRONJ in Oncological and Nononcological Patients Taking or Not Corticosteroid Therapy

**DOI:** 10.1155/2021/7855497

**Published:** 2021-08-25

**Authors:** Paolo Garzino Demo, Alessandro Bojino, Fabio Roccia, Maria Chiara Malandrino, Stefan Cocis, Guglielmo Ramieri

**Affiliations:** Division of Maxillofacial Surgery, Surgical Science Department, Città della Salute e delle Scienze Hospital, University of Turin, Italy

## Abstract

Medication-related osteonecrosis of the jaw (MRONJ) is a severe side effect caused by antiangiogenic antiresorptive drugs used to treat various oncological and non oncological diseases. The clinical and radiological characteristics of MRONJ depend on the type of causative drug, the time of administration, and its dosage. Proven systemic risk factors like anemia, uncontrolled diabetes, corticosteroid therapy, and chemotherapy in neoplastic diseases (e.g., high doses of methotrexate up to 30 mg daily) significantly increase the chances of acquiring MRONJ. The risk factors themselves can affect treatment outcomes. Although the main scientific societies have recently disseminated good practice rules on the patient's prevention, diagnosis, and management, there are still no guidelines on shared therapeutic strategies. In general, if conservative treatment fails, surgical treatment is considered, including local debridement, osteoplasty, and marginal or segmental osteotomy. In literature, cohorts of heterogeneous patients with MRONJ have been analyzed for a long time, resulting in a lack of uniformity of information and difficulties interpreting the data. According to the American Association of Oral and Maxillofacial Surgeons criteria, this retrospective study evaluates the surgical treatment outcomes of 64 patients with stage II-III MRONJ, evaluated at the Department of Maxillofacial Surgery of the University of Turin (Italy). The first objective of this retrospective study is to evaluate treatment results for stages II-III in all cases; the second objective is to evaluate the same results by dividing the sample into different cohorts of patients: first, based on the underlying pathology, i.e., oncological and non oncological, and secondly, based on the drug or combination of drugs they took.

## 1. Introduction

Medication-related osteonecrosis of the jaw (MRONJ) is an adverse drug reaction described as the progressive destruction and death of bone affecting the jaws of patients exposed to the treatment with medications known to increase the risk of disease in the absence of previous radiotherapy [1, 2].

This pathological condition is a side effect of taking various molecules for different pathologies. Consequently, it has an entirely different incidence depending on the original pathology treated and the drugs taken. Indeed, MRONJ develops in about 7-14% of oncologic patients who take high doses of bisphosphonates and denosumab and in 0.01-0.1% of patients with osteoporosis who use bisphosphonates or DNM orally, even at low doses [3–6].

Fewer cases of MRONJ are related to taking antiangiogenic agents, such as antivascular endothelial growth factor (VEGF) monoclonal antibody (bevacizumab), VEGF decoy receptors or VEGF-Trap (aflibercept), or small molecule tyrosine kinase inhibitors (TKI), which block downstream signalling pathways of VEGF (e.g., sunitinib, cabozantinib, and sorafenib) [7, 8], when used alone (i.e., antiresorptive drug-naïve) or in association with antiresorptive drugs [9].

There is no universally recognized protocol for the treatment of MRONJ, in either oncologic or non oncologic patients. In general, if conservative treatment fails, surgical treatment is considered, including local debridement, osteoplasty, and marginal or segmental osteotomy. The healing rates vary depending on the stage of MRONJ [10–13].

To date, no studies have compared the results of surgical treatment between patients stratified by the underlying pathology, i.e., oncological and non oncological, and the type of drug that caused MRONJ as a side effect.

It is now clear that MRONJ has a different radiological and clinical course depending on the type of underlying conditions (e.g., oncological and non oncological).

Proven systemic risk factors like anemia, uncontrolled diabetes, corticosteroid therapy, and chemotherapy in neoplastic diseases (e.g., high doses of methotrexate up to 30 mg daily) significantly increase the chances of acquiring MRONJ. The risk factors themselves can affect treatment outcomes [14, 15].

The first objective of this retrospective study is to evaluate treatment results for stages II-III of MRONJ according to the American Association of Oral and Maxillofacial Surgeons criteria (AAOMS) [2] in all cases.

The second objective is to evaluate the same results by dividing the sample into different cohorts of patients subdivided, both according to the primary pathology, i.e., oncological and non oncological to the drug or combination of drugs taken.

## 2. Material and Methods

### 2.1. Patients

From 2006 to 2018, 148 patients with stage II-III MRONJ were evaluated at the Department of Maxillofacial Surgery of the University of Turin (Italy).

Patients included in this retrospective study had a diagnosis of MRONJ, a life expectancy of over one year, and no contraindication to general anesthesia and had not undergone radiotherapy in the cervical-cephalic district.

The following data were collected: age, sex, underlying diseases, type and duration of antiangiogenic or antiresorptive drugs, corticosteroid and disease-modifying antirheumatic drugs (DMARDs), such as hydroxychloroquine and methotrexate, staging of MRONJ, localization of lesions, local aggravating factors, previous treatment of MRONJ, type of surgical treatment, complications, and outcomes.

In total, 64 of 148 patients met the inclusion criteria and were reported.

The mean age was 68 ± 11.6 years (range 44-89 years); there were 45 females (70.3%) and 19 males (29.7%).

For the study, patients were divided into different cohorts depending on the type of drug taken and the underlying pathology: non oncological patients were treated with antiresorptive drugs and oncological patients were treated with antiresorptive drugs in combination with antiangiogenic agents or treated with antiangiogenic agents alone (Table 1).

**Figure 1:**
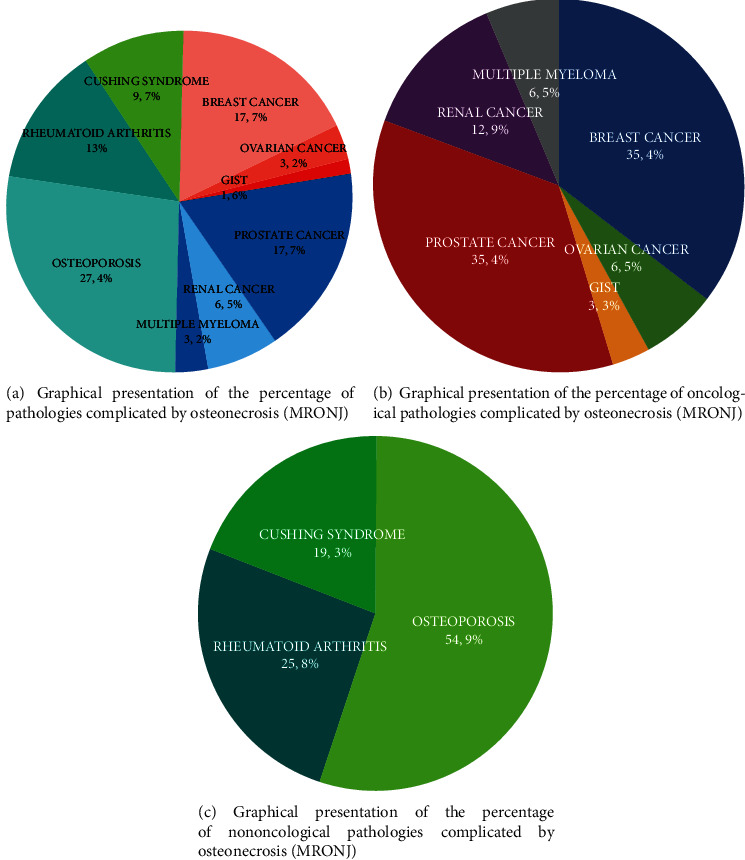
The patients' cohorts (oncologic and nononcologic) and the percentage of patients for any condition.

Figures 1(a)–1(c) summarize the patient cohorts (oncological and nononcological) and the patients' percentage for each condition.

### 2.2. Time of Onset of MRONJ

The time between administration of the drugs and diagnosis of MRONJ was analyzed in the patients' cohorts.

### 2.3. Staging

The authors used the MRONJ staging system of the American Association of Oral and Maxillofacial Surgeons [2].

### 2.4. Surgical Treatments

Treatment for oncologic patients was discussed by an oncologist, a maxillofacial surgeon, and an anesthesiologist. In non oncologic patients, treatment was discussed by an endocrinologist, a maxillofacial surgeon, an internist, and an anesthesiologist.

A total of 64 MRONJ sites were treated surgically (Table 2).

MRONJ surgical procedures have been divided into the following:

- Major surgery marginal (MR) or segmental (SR) resection of the mandible: maxillary defects were classified according to Brown's classification [16]
- Minor surgery (mandibular or maxillary sequestrectomy and curettage)

In mandibular MRONJ, the choice between MR and SR was based on the stage of the disease. Stage II diseases of the mandible involving the alveolar process exclusively and sufficient residual bone stock were treated mainly with MR. Stage III mandibular MRONJ involving the basal process was treated with SR.

All the cases that underwent MR, with sufficient residual bone stock, had primary closure of the surgical site with a tension-free mucoperiosteal flap. In all the MR patients with insufficient bone stock and risk of mandibular fracture, a 2.0 mm plate was placed across the resection site.

In SR patients, mandibular continuity was ensured by a 2.4 mm locking reconstructive plate; intraoral tissue reconstruction and coverage were ensured by a platysma myocutaneous flap, pectoralis major myofascial flap (PMMF), or revascularized free fibular flap (FFF).

For maxillary MRONJ, resection resulting in Brown class I defects was accompanied by functional endoscopic sinus surgery when preoperative imaging showed inflammation of the maxillary sinus. For reconstruction of maxillary defects resulting in class II or higher defects, the buccal fat pad and the tension-free mucoperiosteal flap, the buccinator myomucosal flap, or the temporal myofascial flap were used.

### 2.5. Follow-Up

Clinical follow-up examinations were conducted at 1, 3, 6, 12, and 18 months after treatment. Recorded healing parameters included complete resolution of the MRONJ without signs of bone exposure or infection and disappearance of pain and swelling.

### 2.6. Statistical Analysis

Descriptive statistics were generated for the main quantitative variables; frequency distributions were provided for nominal and ordinal qualitative variables.

The survival curves were calculated using the Kaplan-Meier algorithm. Time zero was defined as the date of the patient's surgery. Patients without recurrence were included only in the total number at risk of recurrence up to the last follow-up. Therefore, the survival rate only changed at the time of recurrence. The calculated survival curve was most likely estimated as the true survival curve. A log-rank test was used to explore the differences between the stratified survival curves for the variable of interest.

**Figure 2:**
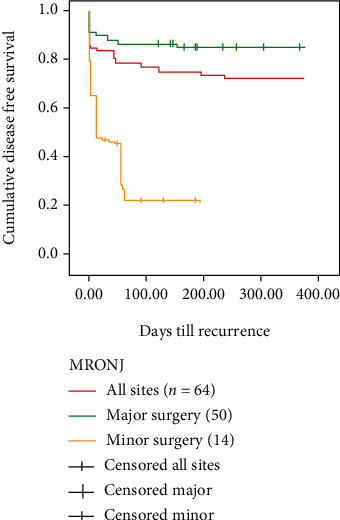
Kaplan-Meier cumulative disease-free survival since resection. There was significant difference between the major and minor surgery.

**Figure 3:**
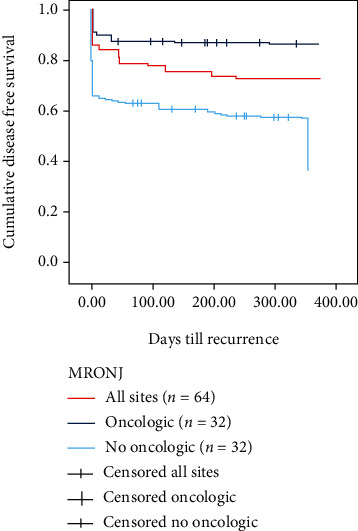
Kaplan-Meier cumulative disease-free survival since resection. There was significant difference between the oncologic sites and nononcologic sites.

The probability of recurrence was calculated using the product-limit method (Kaplan-Meier), with the 95% confidence interval (CI) calculated as 1.96 SE (Figures 2 and 3). The difference in survival between subgroups was tested using log-rank statistics. The level of statistical significance was set with a *p* value < 0.05. The statistical analyses were carried out using the Stata 8 software.

The local ethics committee approved this retrospective study. All patient data were anonymized after extraction from the clinical research databases.

## 3. Results

Thirty-two patients were included in the non oncologic cohorts and were treated with antiresorptive drugs. Among the 32 patients included in the oncological cohort, 19 were treated only with antiresorptive drugs and 13 were treated with antiangiogenetic ones, of which 8 with antiresorptive drugs in association with antiangiogenic agents and 5 with only antiangiogenic agents.

### 3.1. The Time between Administration of the Drugs and Diagnosis of MRONJ

Among the 5 oncological patients treated with only antiangiogenic agents, the time of onset of MRONJ was 6.1-11 months (mean 8.6 months), while for those treated with antiresorptive drugs alone or in combination with antiangiogenic agents, the time of onset was 6.5-16.71 months (mean 9.4 months). Among 19 oncological patients treated only with antiresorptive drugs, the time of onset of MRONJ was 12-34 months (mean 22.7 months).

Among 32 non oncological patients, the time to onset of MRONJ was 20-76 months (mean 24.7 months).

### 3.2. Trigger Events

The most common triggering events of MRONJ were tooth extraction (47.4%), periodontal disease (22%), peri-implantitis (17%), and prosthetic trauma (3.4%); 10.2% of cases were spontaneous.

In non oncologic patients with bone-targeted agents related to ONJ, tooth extraction was the most frequent etiological event (62.5%), followed by periodontal disease (21.9%), implantology (9.4%), and prosthetic trauma (6.2%); among the 24 sites of the oncologic patients with BTA- or BTA plus anti-angiogenic-related BRONJ, the most common etiological event was tooth extraction (33%), followed by implantology (29%). No causal event was noted in the 5 anti-angiogenic-related ONJ sites.

### 3.3. Staging

In the mandible, 18 out of 43 (41.9%) of MRONJ sites involved the alveolar process above the inferior alveolar nerve canal (stage II), and 25 out 43 (58.1%) of MRONJ sites were extended below the inferior alveolar nerve canal (stage III).

In the maxilla, 6 out of 21 (28.6%) of MRONJ sites involved only the alveolar process (stage II), while 15 out of 21 (71.4%) were extended to the maxillary sinus (stage III).

### 3.4. Surgery

All the 64 sites were surgically treated, and complete recovery was achieved in 75%, with persistence or recurrence occurring in 25%.

Considering the location of the MRONJ, complete healing in the maxilla was achieved in 90.9% of stage II disease and 75% of stage III treated with major surgery; complete recovery for the mandible localizations treated with major surgery was achieved in 100% of stage II cases and 80% in stage III cases with statistically significant difference between the two different locations (*p* value < 0.05). Major surgery achieved complete healing in 90% of 50 stage II-III MRONJ sites. 14 stage II sites were treated with minor surgery: 6 in the maxilla and 8 in the mandible. Among them, complete healing was achieved in 3 sites (21.4%). Statistical analysis of the data showed that major surgery was more likely to achieve complete healing than minor surgery (*p* < 0.001) (Table 3).

Surgical complications included 11 patients (24.4%) with mucous dehiscence, 10 (22.2%) with generalized pain, 8 (17.8%) with hematoma, and 16 (35.6%) with paresthesia/anesthesia of the IANC.

Considering the surgery results by dividing patients treated into oncological and non oncological patients, oncological patients had a healing rate of 93% while nononcologic ones had 69.4% (*p* < 0.05).

The 14 non oncologic patients of whom 8 were treated with antiresorptive drugs and corticosteroids (rheumatoid arthritis) and of whom 6 with Cushing's syndrome were treated with antiresorptive drugs had a cure rate of 39.9%.

**Figure 4:**
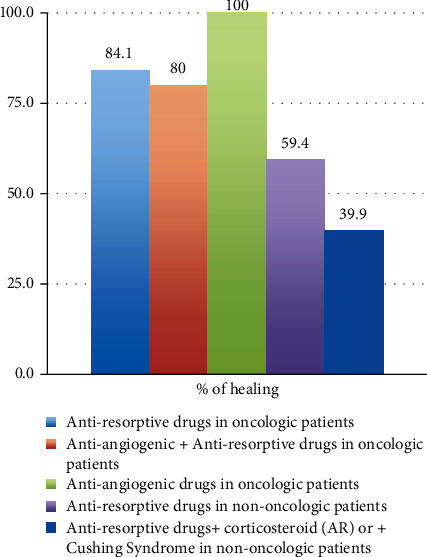
The percentage of healing depending on the drug taken after surgery.

The 19 oncologic patients treated with antiresorptive drugs had a cure rate of 84.1%; the 7 oncologic patients who took antiangiogenetic and antiresorptive drugs have a cure rate of 80%. The 5 oncologic patients who took only antiangiogenic agents had a cure rate percentage of surgical therapy of 100% (Figure 4).

## 4. Discussion

In the medical history of MRONJ patients, there is always a past or current pharmacological intake of an antiresorptive agent or, less commonly, of an antiangiogenic agent. The efficacy of bisphosphonates and their relative safety have implemented their use since 2001 in various clinical diseases, particularly for the control of metastatic bone lesions in oncologic patients and the prevention of fractures associated with postclimacteric osteoporosis. Alongside antiresorptive drugs, in combination or as exclusive therapy in oncologic patients, antiangiogenetic agents are more recently used and responsible for an overall lower number of cases but increasing in recent years [17].

The main factors that seem to increase the risk of MRONJ have been identified (drug used, exposure time, duration of therapy, method of administration, comorbidities, and local disorders), and the primary scientific societies have recently disclosed rules of good behaviour in the field of patient prevention, diagnosis, and patient management. However, there are still no guidelines on therapeutic strategies to be adopted [5]. Furthermore, in the literature, cohorts of heterogeneous patients have been analyzed for a long time, lacking uniformity in information and having difficulties in interpreting data [5]. There are substantial differences in MRONJ epidemiology and treatment outcomes between oncologic and non oncologic patients in the literature. Some of these data are already present but dispersed in inhomogeneous studies combining different types of MRONJ.

In this study, the authors analyzed MRONJ stage II and III patients' surgical outcome to evaluate the study population's characteristics by dividing the sample into oncologic and non oncologic patients and by type of therapy. The time between drug administration and the onset of MRONJ differed depending on the type of drug taken. In the cohort of oncologic patients treated only with antiangiogenic agents, the meantime to onset was very short (6.1-11 months). In oncologic patients treated with BTA, alone or in combination with antiangiogenic agents, the time to onset was similar (6.5-16.71 months).

These data are entirely in line with data from a recent multicentre retrospective study [18]. However, in the cohort of non oncologic patients treated with BTA, the time of onset was significantly longer at 45.90-59.58 months, compared to oncologic patients [19].

The responsible trigger event differs in the two groups. In this case series, the responsible trigger event for the both groups was tooth extraction (32-33%). In non oncologic patients, the second triggering event is periodontal disease (21.9%), while in oncologic patients with ONJ related to BTA intake and ONJ associated with concomitant BTA antiangiogenic intake, the second trigger was found to be the implantology (29%). This could be explained by many dentists' tendency to avoid implantology in osteoporotic patients who have poor quality and reduced bone quantity. At the same time, they are more willing to perform implantology in patients with tumours outside the oral cavity despite antiresorbent/antiangiogenic therapy. A small percentage of cases were spontaneous (12.5%) and had no triggers. In patients with MRONJ on antiangiogenic therapy, 100% of cases were spontaneous. Inhibition of MRONJ-induced recovery from non-BTA-related drugs probably outweighs the osteoclastic bone remodelling effect typical of BTAs [20, 21].

Although there are many publications on MRONJ management techniques, there is currently no consensus on an ideal treatment protocol. Most specialists use treatment protocols based on the stage of the disease [2]. However, there is no evidence that MRONJ (II and III) advanced stages respond to the different proposed therapies. Furthermore, there is no evidence of the treated lesions' prognosis and eventual recurrence [22]. Early treatment reports by then-called BRONJ reported the impossibility of achieving recovery with surgical therapy, which led to the supposition that patients should undergo palliative and conservative therapies rather than pursue a complete recovery with more aggressive interventions [10]. Still, in 2014, the American Society of Maxillofacial Surgery appeared cautious in recommending the therapeutic approach favouring conservative treatment and leaving surgery a secondary role, preferring minor surgery and limiting it to stage III of the disease [2].

Conversely, a 2014 systematic review showed lower healing results for patients treated conservatively, good results when treated with minor surgery, and better results when treated with extensive surgery, regardless of the stage of a disease considered [10]. Subsequent works agreed with this vision by promoting surgery and considering it more effective at healing the mucous membranes and controlling the disease's progression, especially in the more advanced stages, and more effective at leading to the histological confirmation of the clinical diagnosis of MRONJ [11]. In this retrospective study, major surgery achieved complete healing in 90% of 50 stage II-III MRONJ sites, regardless of the underlying nononcological or oncological disease; at the same time, conservative surgery for MRONJ stage II at multiple sites achieved complete healing in only 21.4% of patients. The difference in healing was statistically significant (*p* < 0.001); the Kaplan-Meier curve for cumulative disease-free survival from resection (Figure 2) also demonstrates the effectiveness of major surgery. These data agree with El-Rabbany et al. who state that surgical treatment results are significantly better than those of medical therapy in advanced stages of the disease (II and III) [23]. Comas-Calonge et al. compared conservative and surgical treatment lines (sequestrectomies, surgical debridement, and bone osteotomies): this study concluded that surgical treatment leads to good result rates from 58% to 100% [24]. To date, no studies have compared the results of surgical treatment in patients stratified by the underlying oncological and non oncological disease and the type of drug that caused MRONJ as a side effect. Considering the surgery results and by dividing the treated patients into oncological and non oncological patients, it can be stated that cancer patients had a cure rate of 93% while non oncological patients had a cure rate of 69.4% (*p* < 0.05).  Figure 3 shows the significant difference in results of surgery in oncologic and non oncologic patients.

Furthermore, by stratifying the surgical therapy results based on the drug taken, a different healing percentage is associated with a different type of drug. From the analysis of this cohort of patients, it is observed that oncologic patients who took antiresorptive drugs have a cure rate of 84.1%, while non oncologic patients who took antiresorptive drugs had a cure rate of 69.4%; patients who have taken antiresorptive drugs and corticosteroids to treat rheumatoid arthritis or those with Cushing's syndrome taking antiresorptive drugs have a 39.9% cure rate; patients who have taken antiangiogenic and antiresorptive drugs have a cure rate of 80%; patients who took antiangiogenic therapy had a 100% cure rate.

These data lead to 5 conclusions: the first is that the results do not seem homogeneous and realistic when combining oncological and non oncological patients; therefore, it seems reasonable to consider them in two separate cohorts; the second is that the clinical and radiological characteristics and MRONJ therapy results vary according to the drug taken, the reason for which it is taken, and its route of administration. These data are not taken into consideration even in the most recent meta-analyses [22, 23, 25, 26]. The third is that, despite the sample's numerical limit under analysis, it is assumed that ONJ related to nonantiresorptive drugs has a better cure rate than BTA-related osteonecrosis of the jaw [27]. The shorter half-life of nonantiresorptive agents [20] could positively affect postsurgical healing [17]. The fourth is that endogenous cortisol production or the intake of corticosteroids combined with BTAs complicates surgical healing in advanced stages [14, 15]. The fifth is that, in light of the results of this study, it seems reasonable to prefer invasive surgery for advanced stages of the disease (II and III), taking into account the average life expectancy of the patient and the impact this therapy would have on the subject's quality of life.

## Figures and Tables

**Table:** (a) Oncologic patients

Therapy	Patients	Indications	Type of administration	Dose/frequency	Time on setting of MRONJ
Zoledronate	12	Metastatic cancer	e.v.	4 mg/3-4 weeks	15-20 months
Pamidronate	8	Metastatic cancer	e.v.	90 mg/every 30 days	24-32 months
Ibandronate	6	Metastatic cancer	e.v.	6 mg/3-4 weeks	22-30 months
Ibandronate	3	Metastatic cancer	o.a.	50 mg/day	27-34 months
Multiple types of BPS	3	Multiple myeloma	o.a., i.m., o.a.	Risedronate 5 mg/day Clodronate 800 mg/day Alendronate 10 mg/day	12-18 months
Tot.	32				

**Table:** (b) Nononcologic patients

Therapy	Patients	Indications	Type of administration	Dose/frequency	Time on setting of MRONJ
Clodronate	6	OS	e.v.	300 mg/day every 3 weeks	38-72 months
Alendronate	13	OS	o.a.	70 mg/week	50-76 months
Denosumab	13	OS/AR/CS	s.c.	60 mg/6 months	20-30 months
Tot.	32				

**Table 2:** Number of MRONJ sites studied in relation to basic oncological and nononcological pathologies.

Nononcologic conditions	Patients
Osteoporosis (OS)	18
Rheumatoid arthritis (AR)	8
Cushing syndrome (CS)	6
Tot.	32
Oncologic conditions	
Breast cancer	11
Ovarian cancer	2
Gist	2
Prostate cancer	11
Renal cancer	4
Multiple myeloma	2
Tot.	32
Total sites undergoing surgery	
	64

**Table 3:** Healing percentages according to the MRONJ site and the extent of the intervention.

	Site	Type of surgery (*N*)	Healing *N* (%)	Tot. healing *N* (%)
Minor surgery	Maxilla	Sequestrum removal/curettage (6)	2 (33.3%)	3 (21.4%)
	Mandible	Sequestrum removal/curettage (8)	1 (12.5%)	

Major surgery	Maxilla	Stage II Alveolar resection (12)	10 (90.9%)	45 (90%)
		Stage III Marginal resection+FESS (4)	3 (75%)	
	Mandible	Stage II Marginal resection (20)	20 (100%)	
		Stage III Segmental resection (16)	12 (80%)	

## Data Availability

The data used to support the findings of this study are available from the corresponding author upon request.
