# Corrigendum to “Different Presentation and Outcomes in the Surgical Treatment of Advanced MRONJ in Oncological and Nononcological Patients Taking or Not Corticosteroid Therapy”

**DOI:** 10.1155/2021/9795102

**Published:** 2021-11-20

**Authors:** Paolo Garzino-Demo, Alessandro Bojino, Fabio Roccia, Maria Chiara Malandrino, Stefan Cocis, Guglielmo Ramieri

**Affiliations:** Division of Maxillofacial Surgery, Surgical Science Department, Città Della Salute e Delle Scienze Hospital, University of Turin, Italy

In the article titled “Different Presentation and Outcomes in the Surgical Treatment of Advanced MRONJ in Oncological and Nononcological Patients Taking or Not Corticosteroid Therapy” [1], there are a number of errors in the values of Table 3 in “Results” as identified by the authors following the publication of the article. The corrected Table 3 is as below, and the authors confirm that this does not impact the conclusions of the article.

Additionally, paragraphs 1 and 2 in “Surgery” found under the “Results” should be corrected as follows:

“All the 64 sites were surgically treated, and complete recovery was achieved in 72%, with persistence or recurrence occurring in 28%.

Considering the location of the MRONJ, complete healing in the maxilla was achieved in 90.9% of stage II disease and 75% of stage III treated with major surgery; complete recovery for the mandible localizations treated with major surgery was achieved in 100% of stage II cases and 80% in stage III cases with statistically significant difference between the two different locations (*p*value < 0.05). Major surgery achieved complete healing in 86% of 50 stage II-III MRONJ sites. 14 stage II sites were treated with minor surgery: 6 in the maxilla and 8 in the mandible. Among them, complete healing was achieved in 3 sites (21.4%). Statistical analysis of the data showed that major surgery was more likely to achieve complete healing than minor surgery (*p* < 0.001) (Table 3).”

The sentence beginning with “In this retrospective study, major surgery achieved complete healing…” in the “Discussion” should be corrected as follows:

“In this retrospective study, major surgery achieved complete healing in 86% of 50 stage II-III MRONJ sites, regardless of the underlying nononcological or oncological disease; at the same time, conservative surgery for MRONJ stage II at multiple sites achieved complete healing in only 21.4% of patients.”

## Figures and Tables

**Table 3:** 

	Site	Type of surgery (*N*)	Healing *N* (%)	Tot. healing *N* (%)
Minor surgery	Maxilla	Sequestrum removal/curettage (6)	2 (33.3%)	3 (21.4%)
	Mandible	Sequestrum removal/curettage (8)	1 (12.5%)	
Major surgery	Maxilla	Stage II alveolar resection (12)	10 (90.9%)	43 (86%)
		Stage III marginal resection+FESS (4)	3 (75%)	
	Mandible	Stage II Marginal Resection (18)	18 (100%)	
		Stage III segmental resection (16)	12 (80%)	
